# Clinical Outcomes of Stereotactic Body Radiotherapy for Patients with Lung Tumors in the State of Oligo-Recurrence

**DOI:** 10.1155/2012/369820

**Published:** 2012-07-11

**Authors:** Tetsuya Inoue, Norio Katoh, Rikiya Onimaru, Hiroki Shirato

**Affiliations:** Department of Radiology, Hokkaido University Graduate School of Medicine, North 15 West 7, Kita-ku, Sapporo 060-8638, Japan

## Abstract

We retrospectively evaluated the clinical outcomes of patients with oligometastatic lung tumors who underwent stereotactic body radiotherapy (SBRT). Twenty-two patients with one or two oligometastatic lung tumors were treated with SBRT at our institution between 1999 and 2009. With a median follow-up period of 25 months from the date of SBRT to the detection of oligometastatic lung tumors, the patients' 3- and 5-year overall survival (OS) and progression-free survival (PFS) rates were 72% and 54%, respectively. The median disease-free interval (DFI) between the treatment of the primary site and SBRT to oligometastatic lung tumors was 41 months. The OS of patients with a DFI ≥ 36 months was significantly longer than that of the patients with a DFI < 36 months by the log-rank test (*P* = 0.02). For patients with a DFI ≥ 36 months, the 3- and 5-year OS rates were both 88%, compared to 50% for the patients with a DFI < 36 months. The primary tumor of all patients was locally controlled when SBRT to oligometastatic lung tumors was performed, and thus they were in the state of “oligo-recurrence.” Patients with oligometastatic lung lesions treated by SBRT had good prognoses. This was especially true of the patients with a long DFI and in the state of “oligo-recurrence.”

## 1. Introduction

Most patients who have had any recurrent or metastatic sites of cancer are considered to be in their last stage of life. However, new notions of oligometastases and oligo-recurrence have been proposed [1–9]. Oligometastases is the state in which the patient shows distant recurrence in only a limited number of regions. The clinical state of oligometastatic disease was proposed in 1995 by Hellman and Weichselbaum [1], who hypothesized that local control of oligometastases may yield improved systemic control and prolonged survival. Niibe et al. also discussed the state of oligo-recurrence [2–4]; they defined it as oligometastases with a controlled primary cancer site.

Stereotactic body radiotherapy (SBRT) with a high local dose has been applied to extracranial diseases such as peripheral stage I nonsmall cell lung cancer (NSCLC), and it has been reported to provide excellent local control and survival compatible with surgery [10, 11]. SBRT has also been used in Japan for patients with fewer than three lung metastases ≤5 cm in diameter. In the present study, we retrospectively analyzed our experience with SBRT for patients with oligometastatic lung tumors.

## 2. Methods and Materials

### 2.1. Patient Characteristics

A database of patients who received SBRT for metastatic lung tumors at our institution was used for the patient selection. There were 22 patients who had one or two oligometastatic lung tumors at the time of SBRT and had been treated with SBRT between 1999 and 2009. The diagnosis of the oligometastatic lung tumors was based on whole-body computed tomography (CT). Fluoro-deoxy-glucose (FDG)-positron emission tomography (PET) was performed as needed. The primary tumor of all patients was locally controlled when SBRT to the oligometastatic lung tumors was performed. The treatment methods for the primary sites were surgery in 13 patients and definitive radiotherapy in nine. Definitive radiotherapy consisted of conventional radiotherapy in one patient, brachytherapy in one patient, and SBRT in seven.

We labeled the treatment interval time from the primary sites to oligometastatic lung tumors as the disease-free interval (DFI). In this study, all analyses started from the day of SBRT to oligometastatic lung tumors.

The patient characteristics are given in Table 1. There were 8 men and 14 women, and the median age was 67 years (range 30–84 years). The primary cancers consisted of lung cancer (*n* = 9), head and neck cancer (*n* = 4), breast cancer (*n* = 3), colorectal cancer (*n* = 2), genitourinary cancer (*n* = 2), thymic cancer (*n* = 1), and skin cancer (*n* = 1). The primary histology consisted of adenocarcinoma (*n* = 13), squamous cell carcinoma (*n* = 4), renal cell carcinoma (*n* = 1), transitional cell carcinoma (*n* = 1), large-cell carcinoma (*n* = 1), malignant melanoma (*n* = 1), and apocrine gland carcinoma (*n* = 1). There were 13 patients who had only one oligometastatic lung tumor and nine patients who had two oligometastatic lung tumors. The median tumor size was 15 mm (range 8–47 mm). No chemotherapy was allowed until tumor progression.

### 2.2. SBRT Technique

All patients received SBRT to oligometastatic lung tumors as the definitive radiotherapy. Nine patients received SBRT using a real-time tumor-tracking radiotherapy (RTRT) system, and 13 patients received SBRT without RTRT.

The RTRT system has been described in detail elsewhere [12, 13]. In brief, 1.5 to 2.0 mm gold markers were implanted near the tumor by means of image-guided procedures. CT scans were taken with the patients holding their breath at the end of normal expiration. The gross tumor volume (GTV) was contoured in axial CT images. The clinical target volume (CTV) was defined three-dimensionally as the GTV on CT with a 5 mm margin for metastatic lung tumors and was considered to be equal to the internal target volume (ITV). The planning target volume (PTV) was three-dimensionally defined as the CTV plus a 5 mm margin with optimal reduction near the organ at risk (OAR).

SBRT without RTRT was described as follows. To determine the ITV margin, CT scans were performed three times, with breath holding at the expiratory and inspiratory phases and with free breathing. The three GTVs on CT at three phases were superimposed on the radiation treatment system to represent GTV + ITV. The CTV was defined three-dimensionally as the GTV + ITV on CT with a 5 mm margin. The PTV was three-dimensionally defined as the CTV plus a 5 mm margin with optimal reduction near the OAR.

We administered 48 Gy in four fractions at the isocenter calculated by Clarkson algorism or 40 Gy in four fractions to the 95% volume of PTV by superposition algorism with a treatment period of 4 to 7 days. Patients were treated with 4- or 6-MV photons. SBRT was delivered using multiple noncoplanar static ports.

### 2.3. Followup after SBRT

Follow-up visits were usually every 3 months after SBRT. CT scans were usually performed every 3–6 months after SBRT. Local progression was diagnosed on the basis of histologic confirmation or enlargement of the local tumor on CT that continued for at least 6 months. FDG-PET was recommended when local recurrence was suspected, but this was not mandatory.

### 2.4. Ethical Considerations

Written informed consent to receive SBRT was obtained from all patients. This retrospective study was performed in accordance with the 1975 Declaration of Helsinki, as revised in 2000.

### 2.5. Statistical Analysis

The overall survival (OS) and progression-free survival (PFS) rates were calculated from the date of SBRT to oligometastatic lung tumors using the Kaplan-Meier method. The log-rank test was used to identify significant differences. R version 2.14.2 with the survival packages (R project for statistical computing, Vienna, Austria) was used for the statistical analyses. A value of *P* < 0.05 was considered significant.

## 3. Results

### 3.1. Survival

**Figure 1:**
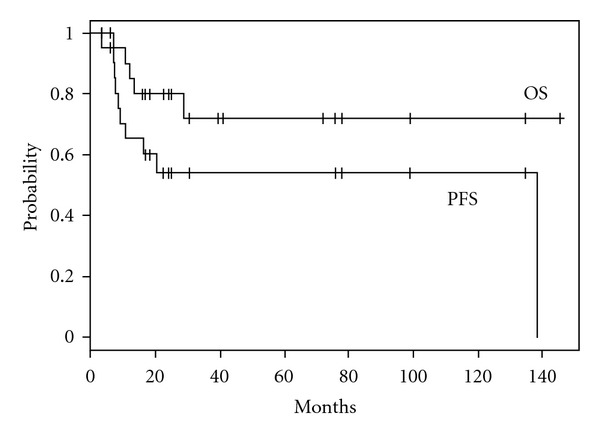
Kaplan-Meier actuarial overall survival (OS) and progression-free survival (PFS) rates.

With a median follow-up period of 25 months (range 4–146 months) from the day of SBRT to oligometastatic lung tumors, the 3- and 5-year overall survival and progression-free survival rates were 72% and 54% (Figure 1). The median DFI between the treatment of the primary site and SBRT to oligometastatic lung tumors was 41 months. The primary tumor of all patients was locally controlled when SBRT to oligometastatic lung tumors was performed; the patients were thus in the state of “oligo-recurrence.”

### 3.2. Patterns of Failure

Disease progression was observed in nine patients (Table 2). All irradiated lesions by SBRT were controlled. New intrapulmonary metastases were observed in four patients, bone metastases were observed in one patient, and liver metastases were observed in one patient. Multiple metastatic lesions including regional lymph node, brain, bone and/or liver were observed in three patients.

### 3.3. Toxicities

Adverse effects were graded according to the Common Toxicity Criteria for Adverse Events, version 3.0. Grade 2. Intercostal neuralgia occurred in one patient. No radiation pneumonitis of grade 3 or more was observed.

### 3.4. Prognostic Factors

**Figure 2:**
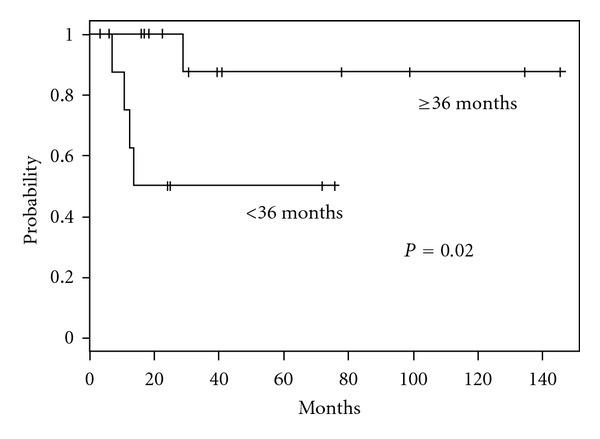
Kaplan-Meier curve of overall survival rates for patients with a disease-free interval (DFI) <36 months (*n* = 9) or ≥36 months (*n* = 13). The groups' survival rates differed significantly (*P* = 0.02).

We also analyzed the survival differences stratified by DFI duration. DFI duration was divided into <36 or ≥36 months. The OS of patients with a DFI ≥ 36 months (*n* = 13) was significantly longer than the OS of those with a DFI < 36 months (*n* = 9) (*P* = 0.02). For patients with a DFI ≥ 36 months, the 3- and 5-year OS rates were both 88%, compared to 50% for patients with a DFI < 36 months (Figure 2).

## 4. Discussion

In this patient population, the 3- and 5-year overall survival and progression-free survival rates were 72% and 54%, respectively, which was equivalent to or better than those in previous studies of oligometastatic lung tumors as follows. Norihisa et al. reported the results of SBRT for oligometastatic lung tumors [14]. The OS rate and PFS rates at 2 years were 84.3% and 34.8%. Rusthoven et al. recently reported the results of multi-institutional phase I/II trials of SBRT for lung metastases [15]. The actual local control rates at 1 and 2 years after SBRT for oligometastatic lung tumors were 100% and 96%, respectively, and the median survival time was 19 months.

A landmark study of more than 5,000 patients by the International Registry of Lung Metastases (IRLM) demonstrated that long-term survival can be achieved in a proportion of patients with lung metastases treated with metastasectomy [16]. The actuarial survival after complete metastasectomy was 36% at 5 years. With the exclusion of the apparently favorable tumors, the survival outcome at 2 years was approximate 70%.

We previously reported the clinical outcomes of stereotactic brain and/or body radiotherapy for patients with oligometastatic lesions. The organs affected by oligometastatic lesions were the brain, lung, and/or adrenal gland [17]. For patients with oligometastatic lung disease, the 3- and 5-year OS rates were both 63%, significantly better than the 22% and 14% of those with brain/adrenal metastases.

In the present study, the DFI between the treatment of primary site and SBRT to oligometastatic lung tumors was the prognostic factor. Norihisa et al. also reported that patients with a longer DFI had a greater overall survival rate [14]. Patients with a DFI ≥ 36 months had significantly greater OS compared to those with a DFI < 36 months. In the IRLM study, a multivariate analysis revealed that a DFI longer than 36 months is a factor associated with improved survival [15]. In our previous study, we also found that patients with a DFI ≥ 12 months had significantly greater OS compared to those with a DFI < 12 months [17].

The IRLM study and multi-institutional phase I/II trials by Rusthoven et al. included locally uncontrolled primary tumors, so-called oligometastases [15, 16]. However, in the present study, the primary tumor of all patients was locally controlled when SBRT to oligometastatic lung tumors was performed, that is, in the so-called state of “oligo-recurrence.” Therefore, the present population's outcomes were equivalent or better than those in the previous study of oligometastatic lung tumors. We were also curious about survival differences between patients with and without oligo-recurrence, but all of the patients in this population were in the state of oligo-recurrence. Moreover, in the present study, the median DFI between the treatment of the primary site and the SBRT to oligometastatic lung tumors reached 41 months, a very long period compared with other studies. However, it was difficult in this study to distinguish second primary lung cancers from metastatic lung cancers, and oligometastatic lung tumors from NSCLC might be second primary lung cancers, which may have better prognoses than metastatic lung cancers.

One shortcoming of the present study is the retrospective nature of the analysis. Patients with sufficient medical conditions were probably selected beforehand to receive SBRT. The large number of patients who died within a short period may have masked the possible progression of the disease and local failure. However, it is notable that there was a definite group of patients treated with SBRT for oligometastatic tumors who experienced long survival even with distant metastasis. A large prospective trial is required to establish the precise benefits of SBRT for patients with oligometastatic lung tumors. Our findings suggest that the DFI should be included in the stratification criteria in a prospective randomized trial comparing treatment with and without SBRT.

In conclusion, patients with oligometastatic lung lesions treated by SBRT had good prognoses, especially the patients with a long DFI and in the state of “oligo-recurrence.”

## Figures and Tables

**Table 1:** Patient characteristics (22 patients).

Characteristics	Value
Age (years)	
Median	67
Range	30–84
Gender (*n*)	
Male	8
Female	14
Primary cancer (*n*)	
Lung	9
Head and neck	4
Breast	3
Colorectal	2
Genitourinary	2
Thymic	1
Apocrine gland	1
Primary histology (*n*)	
Adenocarcinoma	13
Squamous cell carcinoma	4
Others	5
Treatment for primary cancer (*n*)	
Resection	13
SBRT	7
Conventional radiation therapy	1
Brachytherapy	1
Number of oligometastatic tumors (*n*)	
1	13
2	9
Tumor diameter (*n*)	
<20 mm	25
21–30 mm	4
>30 mm	2

SBRT: stereotactic body radiotherapy.

**Table 2:** Patterns of disease progression (9 patients).

Pattern	*n*
New pulmonary metastases	4
Liver metastases	1
Bone metastases	1
Multiple metastases	3
